# Cutting Oxygen Production-Related Greenhouse Gas Emissions by Improved Compression Heat Management in a Cryogenic Air Separation Unit

**DOI:** 10.3390/ijerph181910370

**Published:** 2021-10-01

**Authors:** Miroslav Variny, Dominika Jediná, Miroslav Rimár, Ján Kizek, Marianna Kšiňanová

**Affiliations:** 1 Department of Chemical and Biochemical Engineering, Faculty of Chemical and Food Technology, Slovak University of Technology in Bratislava, Radlinského 9, 812 37 Bratislava, Slovakia; domka444@gmail.com (D.J.); m.ksinanova1@gmail.com (M.K.); 2 Department of Process Technique, Faculty of Manufacturing Technologies with a Seat in Presov, Technical University of Kosice, Bayerova 1, 080 01 Presov, Slovakia; miroslav.rimar@tuke.sk (M.R.); jan.kizek@tuke.sk (J.K.)

**Keywords:** air separation unit, air humidity, emissions, power consumption, emission factors, heat recuperation, absorption cooler, compressed air dryer

## Abstract

Oxygen production in cryogenic air separation units is related to a significant carbon footprint and its supply in the medicinal sphere became critical during the recent COVID-19 crisis. An improved unit design was proposed, utilizing a part of waste heat produced during air pre-cooling and intercooling via absorption coolers, to reduce power consumption. Variable ambient air humidity impact on compressed air dryers’ regeneration was also considered. A steady-state process simulation of a model 500 t h^−1^ inlet cryogenic air separation unit was performed in Aspen Plus^®^ V11. Comparison of a model without and with absorption coolers yielded an achievable reduction in power consumption for air compression and air dryer regeneration by 6 to 9% (23 to 33 GWh year^−1^) and a favorable simple payback period of 4 to 10 years, both depending on air pressure loss in additional heat exchangers to be installed. The resulting specific oxygen production decrease amounted to EUR 2–4.2 t^−1^. Emissions of major gaseous pollutants from power production were both calculated by an in-house developed thermal power plant model and adopted from literature. A power consumption cut was translated into the following annual greenhouse gas emission reduction: CO_2_ 16 to 30 kilotons, CO 0.3 to 2.3 tons, SO_x_ 4.7 to 187 tons and NO_x_ 11 to 56 tons, depending on applied fossil fuel-based emission factors. Considering a more renewable energy sources-containing energy mix, annual greenhouse gas emissions decreased by 50 to over 80%, varying for individual pollutants.

## 1. Introduction

Oxygen is one of the most important technical gases produced worldwide. Oxygen production plants rely on its separation from air by cryogenics [1], membranes [2], or adsorption [3] with the first method being suitable for large-scale production of almost pure oxygen and other gases from air [4,5] the latter two, suitable as mobile oxygen or nitrogen sources are still in development [6,7]. Typical energy consumption in cryogenic units exceeds 200 kWh of electricity per ton of produced oxygen [8,9] and is thus associated with significant environmental impact [1,10]. 

As calculated by Banaszkiewicz and Chorowski [3], minimum thermodynamic work for air separation is around 51 kWh per ton of produced oxygen, which documents the large gap between actual plant performance and minimum energy requirement while highlighting the potential for specific power consumption reduction. At the same time, calculations of vacuum pressure adsorption indicated energy consumption as low as 140 kWh per ton of produced 95% grade oxygen; however, data from real applications [3] show lowest values to be almost three times higher, significantly exceeding those reported from cryogenic units. Calculations performed by Castillo [11] and Markewitz et al. [12] for membrane-based air separation units indicate achievable energy savings of up to 9% compared to conventional cryogenic units. Such systems are especially suitable for oxyfuel power plants. 

Further significant reduction in specific power consumption is expected from thermochemical air separation cycles using perovskite oxides [13] and their powering either by solar irradiation [14] or low-grade heat [15]. They can be further combined with adsorption-based air pre-treatment to reach comparable oxygen (or nitrogen) purity as cryogenics but with a significantly lower carbon footprint [16]. 

Oxygen is widely used in metallurgy [8,17,18], cutting down fuel costs and greenhouse gas emissions [19,20], for oxy-combustion coupled with carbon capture and storage [9,21], production of a variety of chemicals, and in medicine [22,23].

Oxygen production and supply have recently attracted global attention due to the COVID-19 pandemic and the associated significant rise in medicinal oxygen demand [24]. Strained oxygen supplies remain critical nowadays, especially in India and Africa [25,26,27]. Alternative methods of medicinal oxygen production have been tested successfully [28]; however, many hospitals and healthcare facilities rely on a high-percentage oxygen supply from large cryogenic air separation units (ASU). While its supply is in the spotlight of the media, its carbon footprint has received far less attention [29]. A recent study by Balys et al. [30] identifies this knowledge gap and sums up evidence of the environmental impact related to medicinal oxygen supply chain considering 64,800 m^3^ per month medicinal grade oxygen consumption in a hospital. As documented, the global warming potential (GWP) of liquid tank oxygen delivered is around 260 gCO_2_ kg^−1^ while oxygen delivery in cylinders shows much higher environmental impact with a GWP value of over 550 gCO_2_ kg^−1^. In-house oxygen production via pressure swing adsorption shows a somewhat lower GWP value than cylinder delivery but still much higher than liquid oxygen delivery in tanks. These figures fit well with the specific energy consumption values provided above: assuming average power production emission factor of a coal power plant of 0.8 tCO_2_ MWh^−1^ [31] and average specific power consumption of 300 kWh t_O2_^−1^, oxygen emission factor of 240 gCO_2_ kg^−1^ of oxygen is obtained, the difference between this value and the oxygen GWP estimated by Balys et al. [30] can be attributed to oxygen losses and emissions due to oxygen transport and logistics. This stresses the importance of oxygen production energy intensity reduction as the related greenhouse gas emissions represent a large portion of total oxygen supply chain-related emissions. 

### 1.1. Improving the Design and Operation of Cryogenic Air Separation Units

Air separation in cryogenic plants is a well-established technology enabling production of pure gases from air [32,33] with an oxygen production capacity above 5000 Nm^3^ h^−1^ [34]. Its basic layout includes double- or triple-stage air compression with intercooling, compressed air cooling, and removal of residual moisture together with carbon dioxide on a suitable adsorbent [35,36]. Dry, compressed air is then routed to the cryogenic part of the plant, where a single- to triple-column design is usually adopted to obtain one or more gases of over 95 (99) % purity [34]. Thermal coupling of the columns, as well as incorporation of expanders for pressure energy recovery, help reduce the plant’s energy demand [32,37]. Likewise, air pre-treatment by membranes [38,39] or adsorbers [40] can reduce both capital and operational costs of the cryogenic part. 

A major part of the energy demand of a conventional cryogenic unit can be allocated to the compression section due to the need to compress air from atmospheric pressure to typically 500 to 700 kPa [32]. Minor energy consumption of up to 10 to 15% of the total value is associated with adsorbent (air dryer) regeneration. Power consumption in the cryogenic section is negligible and it is associated with process pumps operation and, if process expanders driving generators are integrated in the unit, it can even reach zero. Therefore, most operation optimization studies focus on compression and purification sections looking for possibilities of a power consumption cut, while optimal design studies also include the cryogenic part of the plant. 

Many recent studies have already focused on the reduction in the plant´s carbon footprint, either by improving the design or by deeper integration within industrial clusters [4,40,41]. These studies are often performed in expert simulation software, e.g., Aspen Plus or Aspen Hysys [42,43]. Adamson et al. [5] published a framework for steady state operation optimization of three ASU and the related gas network comprising multiple compressors based on load scheduling achieving electric energy consumption reduction of 5%. Singla and Chowdhury [8] optimized oxygen production costs in an integrated iron and steel mill by ASU´s cryogenic section reconfiguration and oxygen purity adjustment. Compared to the original layout, oxygen production costs were reduced by 15 to 25% in the improved set-up while keeping the single-column plant design. Wang et al. [1] investigated various column heat integration schemes and compared their energy consumption to the conventional plant layout, concluding that a partly heat-integrated design is the most suitable, ensuring carbon emissions cut by over 40% and a reasonable decrease in total annual costs.

Air compression before cryogenic separation results in a significant amount of waste heat that is usually disposed of by water intercoolers and coolers [44]. Rong et al. [45,46] analyzed the possibilities of compression heat recovery and reuse through inlet air cooling and dehumidification. They found that the proposed system, incorporating a desiccant wheel and an organic Rankine vapor compression refrigeration cycle helped reducing the energy costs of a model ASU by 5% with a payback period of around 5 years. Other researchers studied options for compression heat reuse in an oxy-fuel power plant, finding its optimal allocation in fuel pre-drying [39] and feedwater preheating train [4]. Similarly, Escudero et al. modeled integration of an oxy-fuel plant coupled with carbon capture and storage into energy system of a fluid catalytic cracking unit [47]. Air compression heat was partly utilized for low temperature boiler feedwater heating, while compressed air pre-cooling and intercooling was enhanced by compression cooling system installation. Zhou et al. proposed using air compression heat to drive an organic Rankine cycle [48] that in turn drove a compression cooling unit for air pre-cooling, thus reducing the power demand of the model ASU by over 4%. As energy demand for air cooling is tightly interconnected with air properties, especially with its humidity, the effect of its variations on the achievable power consumption reduction is of key importance but remains largely unexplored.

### 1.2. Greenhouse Gas Emissions Attributable to ASU Operation

Air separation units consume electric energy produced either on-site (if integrated in an oxyfuel power plant) or elsewhere, which is more common. Making the operation of an ASU more efficient thus means cutting down its greenhouse gas (GHG) emissions. Carbon dioxide emissions are considered as the most relevant ones in this regard and a lively debate on the correct attitude towards calculation and evaluation of its emission factors is in progress [49,50]. It is generally agreed that marginal emission factors (MEF) should better represent the real impact of power consumption change on the related CO_2_ emissions [51,52]. For even more precise carbon accounting for processes with very variable power demand, daily or even hourly MEF are recommended to be applied [53]. Several analyses attempted to assign energy consumption and the associated GHG emissions to industrial branches [54] and society spheres [55] and to formulate suitable emission factors predictions for decades ahead [56,57]. Even though power production becomes gradually cleaner as advanced techniques and flue gas cleaning systems are adopted in thermal plants and old plants are ruled out of service [58], MEF values were agreed to be highly spatiotemporally specific. Thus, recent studies employing the MEF always relate them to a specific period and country [52,59], thus requiring reliable structural data about power sources and transmission system operation [60]. A recent extensive review by Hamels et al. [49] evaluating over 100 related studies revealed both the absence of a unified approach to the estimation of GHG emissions related to power production and consumption and the significant variability of individual emission factors.

### 1.3. Aims of the Study

As results from the performed literature survey, intense research on optimal design and operation of cryogenic ASU is in progress worldwide. Especially for standalone ASUs it is imperative to exploit all possibilities for power consumption reduction offered by heat recuperation and reuse. Although the possibility of using compression heat has been analyzed in recent studies, the effect of inlet air humidity has to be studied further. The associated cut in power consumption and, thus, in GHG emissions should be clearly quantified. However, no unified approach to this topic has been presented up to now. Instead of using a single power emission factor, applying a range of its values yields more realistic, even if less precise, results. 

To fill the identified knowledge gap, the following aims are pursued in our study:

- Analyzing possible power consumption reduction by using compression heat for air pre-cooling and intercooling via absorption coolers (ACH);
- Investigating the effect of air cooling on energy consumption for adsorptive air dryer regeneration and evaluating the associated impact of variable air humidity using measured hourly data;
- Estimating basic economic parameters of ACH installation in a model ASU;
- Analyzing and quantifying achievable reduction in carbon dioxide, nitrogen oxides, sulfur oxides, and carbon monoxide by applying various emission factors reported in literature as well as those obtained by modeling of an industrial thermal power plant operation.

The study method includes modeling of an ASU with basic layout and parameters adopted from [36] in Aspen Plus^®^ (Aspen Plus V 11, Aspen Technology Inc., Bedford, MA, USA),

- starting with basic design and verification of the values,
- introducing model changes, incorporating adsorption coolers and cooling water towers,
- testing the sensitivity of energy savings by proposed technology changes to frictional pressure losses and analyzing energy consumption for adsorptive air dryers’ regeneration.

Subsequently, a set of measured ambient air properties data in form of hourly averages from one-year period was used to recalculate the achievable power consumption cut to estimate a simple payback period of the proposed ACH installation. In the end, an industrial thermal power plant model was set up considering publicly available data on the associated emissions of air pollutants, yielding power emission factors for carbon dioxide, nitrogen oxides, sulfur oxides, and carbon monoxide. The obtained values were compared with data available in relevant literature and by their implementation, a range of achievable cuts in GHG emissions was calculated for every considered pollutant. Thereby a complex energetic–economic–environmental view on oxygen production in cryogenic ASUs is provided.

## 2. Materials and Methods

### 2.1. Air Separation Unit Model

**Figure 1:**
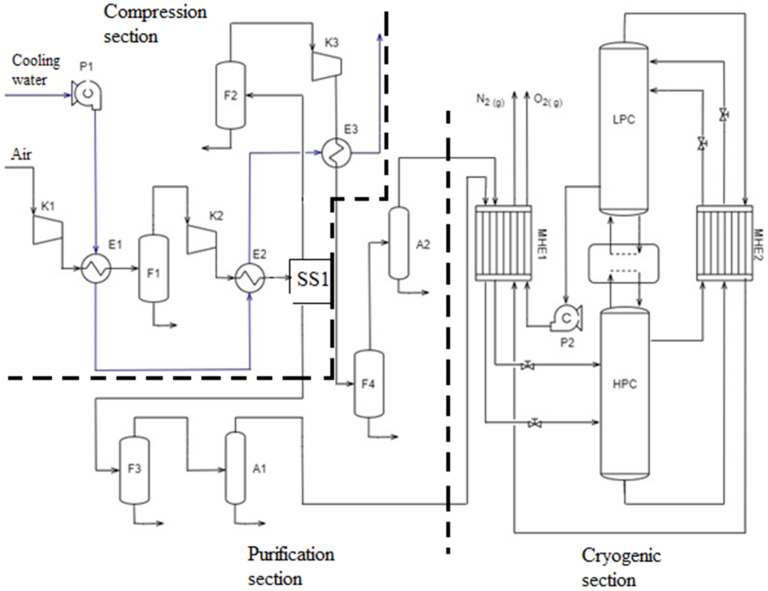
Simple flow diagram of two-column cryogenic air separation model design C1, adapted from [36]. A1,2: adsorbers; E1-3: heat exchangers; F1-4: phase separators; HPC: high-pressure column; K1-3: compressors; LPC: low-pressure column; MHE1,2: multi-stream heat exchangers; P1,2: pumps; SS1: stream splitter. Dashed lines = borders of unit´s sections.

A basic cryogenic ASU model is completely based on a study by Hamayun et al. [36] which aimed to identify the most suitable two-column cryogenic air separation unit configuration by evaluating seven alternatives based on exergy analysis. For each alternative configuration of two-column cryogenic air separation unit, a rigorous mathematical model was developed and simulated using Aspen Plus^®^ V10 (Aspen Technology Inc., Bedford, MA, USA). The results showed that the design alternative labelled as “C1” was the most exergy-efficient for cryogenic air separation, and therefore this design alternative was used to develop the basic mathematical model in program Aspen Plus^®^ V11 in the current study. A simple flow diagram of two-column cryogenic ASU model design (C1) is presented in Figure 1.

The model consists of three main sections: compression, purification, and cryogenic. Compression section consists of three compressors (K1, K2, K3), water cooled intercoolers (E1, E2, E3) and flash separators (F1, F2, F3 and F4) for condensed water removal from cooled compressed air. After the second compression stage, compressed air is separated into two streams with optimal mass ratio of 1:2 as discussed in [36]. Purification section consists of two adsorbers (A1, A2), where residual water vapor and carbon dioxide are removed from compressed air by adsorption before entering the cryogenic section. Cryogenic section is composed of two multi-stream heat exchangers (MHE1, MHE2), where air is cooled and liquified using product streams from the distillation section and two distillation columns—high pressure column (HPC) and low-pressure column (LPC). The columns are heat-integrated in a standard way for two-column cryogenic distillation—the condenser duty of the HPC column equals the reboiler duty of the LPC column.

All input model parameters of inlet material streams, main process equipment, thermodynamic model standard Peng–Robinson and other assumptions were adopted from [36], including the following assumptions and simplifications:

- Steady state;
- Isentropic efficiency of compressors: 72%;
- Isentropic efficiency of pump: 80%;
- Pressure loss in all heat exchangers: 10 kPa;
- Outlet air temperature in water coolers: 40 °C;
- Number of stages in HPC column: 60;
- Position of feed stages in HPC column: 50 and 60;
- Number of stages in LPC column: 60;
- Position of feed stages in LPC column: 1 and 42;
- Total pressure loss in HPC column: 20 kPa;
- Total pressure loss in LPC column: 30 kPa;
- Zero heat (and cold) losses from all modeled process equipment surfaces.

Atmospheric air is the main inlet raw material stream. Further model specifications are provided in Appendix A, including properties of air (Table A1) and cooling water (Table A2). Each process equipment of the selected cryogenic ASU design was modeled by the most suitable model available in Aspen Plus^®^ V11. An overview of selected models and required input model parameters is shown in Appendix A, Table A3.

### 2.2. Process Flow Diagram of Cryogenic Air Separation Unit

**Figure 2:**
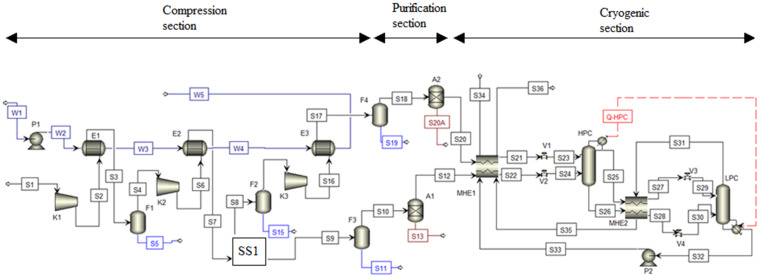
Process flow scheme of developed model in Aspen Plus^®^ V11. A: adsorber; E: heat exchanger; F: phase separator; HPC: high-pressure column; K: compressor; LPC: low-pressure column; MHE: multi-stream heat exchanger; Q: heat flow; P: pump; S: material stream; SS1: stream splitter; V: valve; W: cooling water stream.

A process flow diagram presents a graphical form of the developed mathematical model and relates to the basic model of cryogenic ASU described in previous text and is provided in Figure 2.

Critical analysis of model parameters showed that the pressure loss in both adsorbers and adsorption heat of water were neglected in [36]. Therefore, two additional adjustments of input model parameters were necessary:

(1) Specification of air pressure loss in adsorbers A1 and A2 according to the pressure loss specified in all modeled heat exchangers = 10 kPa.
(2) Addition of fictive heat exchangers E4 and E5 to the basic model downstream of adsorbers A1, A2 to include the effect of adsorption heat of water. The most suitable value of adsorption heat of 3000 kJ kg^−1^ was specified according to [61].

**Figure A1:**
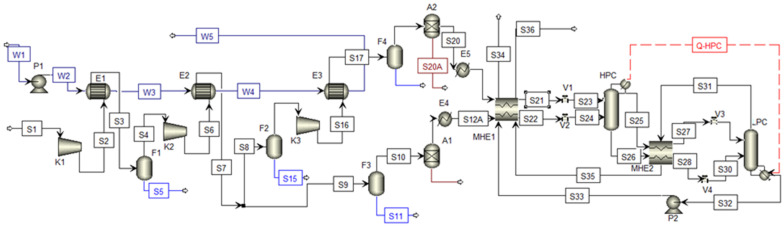
Modified process flow scheme. A: adsorber; E: heat exchanger; F: phase separator; HPC: high-pressure column; K: compressor; LPC: low-pressure column; MHE: multi-stream heat exchanger; Q: heat flow; P: pump; S: material stream; SS1: stream splitter; V: valve; W: cooling water stream.

Mathematical model modified in Aspen Plus^®^ V11 is presented in Appendix A, Figure A1.

### 2.3. Regeneration of Air Dryers

Calculation of power input for adsorbers regeneration was based on referential parameters obtained from available technical documentation of real adsorbers with a similar size [62], assuming that the same means of regeneration were adopted for both referential and model adsorbers (electric heating of inlet air). Approximate electrical power input for model adsorbers regeneration, $Q_{p,model}$, was calculated from known electrical power input for referential adsorber regeneration ($Q_{p,ref}=$ 165 kW) and mass flow of adsorbed water in referential adsorber (${\overset{\cdot }{m}}_{H2O,ref}=$ 102.6 kg h^−1^) and known mass flow of adsorbed water in modeled adsorber, ${\overset{\cdot }{m}}_{H2O,model}$, Equation (1).

(1) $$Q_{p,model}=\frac{{\overset{\cdot }{m}}_{H2O,model}}{{\overset{\cdot }{m}}_{H2O,ref}}\;Q_{p,ref}$$

### 2.4. Model with Absorption Cooling

The basic model of cryogenic ASU shows potential for compression section design improvement. Additional cooling of compressed air before the first compression stage and after the first and second compression stages to temperatures lower than model values can thus be considered. In case of additional cooling of compressed air, more condensed water from air and significant energy savings for adsorbers regeneration due to lower amount of adsorbed water is presumed. Compression work reduction is also expected. 

Two design improvement possibilities were considered:

- Using own (internal) available heat for cold production.
- Using external available heat and cold.

#### 2.4.1. Own (Internal) Heat for Cold Production

All heat content of compressed air was absorbed by cooling water in intercoolers (E1, E2, E3) in the basic technology. The proposal was to use heat potential obtained in hot compressed air stream after the first and second compression stage as a source of energy for hot-water ACH for additional cooling of compressed air before the next compression stage. Thus, reduction in compression work in compressors K2 and K3 due to lower inlet air temperature is achieved. Heat released by ACH is led away by available cooling water. Additional utilization of cooling water offered potential for cooling towers implementation into the basic mathematical model to secure a closed loop cooling water system. 

The most suitable technical parameters of hot-water ACH for the modeled system were obtained from available technical documentation [63] and are presented in Appendix B, Table A4 and Table A5.

The most suitable model parameters for cooling towers were obtained from available technical documentation [64] of real cooling towers for large volumes of water. According to the available technical documentation, four identical cooling towers to cool cooling water back to the input process temperature of 25 °C were designed. Their selected technical parameters are presented in Appendix B, Table A6.

For standard mathematical modeling of cooling towers in Aspen Plus, a RadFrac model with a few equilibrium stages considering neither reboiler nor condenser was used. The RadFrac model calculates the liquid and vapor/gas equilibrium on each equilibrium stage using property package NRTL [65], which was specified for all cooling water systems. Input model parameters of cooling tower are presented in Appendix B, Table A7. Input parameters for air were specified according to input model parameters of inlet air stream S1.

#### 2.4.2. External Heat for Cold Production

The aim of this analysis was to evaluate the possibility of pre-cooling inlet air stream S1 before entering the first compression stage to achieve reduction in compression work of compressor K1 by absorption cooling of inlet air stream to temperatures below 25 °C.

It is assumed that energy required for absorption cooler operation includes available waste, zero cost, heat from a nearby industrial plant. This assumption is justified, as low-grade waste heat, suitable for this purpose, is commonly found in heavy industry (iron and steel, refining, pulp and paper, cement and lime and others). A study by Brueckner et al. [66] revealed that at least 127 PJ per annum (13% to total industrial fuel energy consumption) waste heat is available in German industry with more than 80% at temperatures above 100 °C. Similarly, tens of PJ per annum of recoverable waste heat were reported by Law et al. [67] for the UK industry.

Simultaneously, absorption and condensation heat from ACH is led away by cooling water. Input model parameters of suitable hot-water ACH shown in Appendix B, Table A8, were obtained from available technical documentation of a real ACH [63].

**Figure 3:**
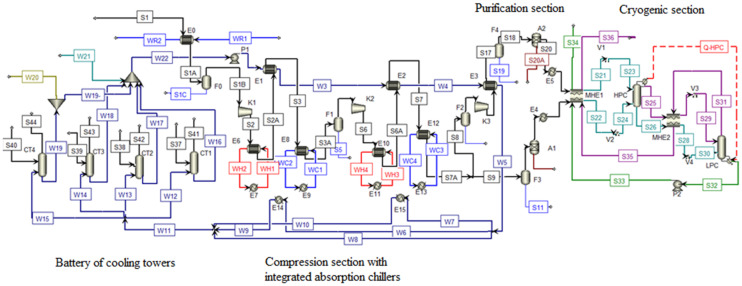
Process flow diagram of mathematical model comprising air pre-cooling and intercooling by absorption cooling equipment. WC: chilled water, WH: hot water, WR: return water.

Process flow diagram of mathematical model comprising air pre-cooling and inter-cooling enhanced by absorption cooling is presented in Figure 3. ACH and the necessary new heat exchangers are included as follows: E14, E15—water cooling of ACH absorbers and condensers; E0, E8, E12—air coolers utilizing cooled water from ACH; E7, E11—ACH generators; E6, E10—hot compressed air coolers for hot water production for ACH.

### 2.5. Ambient Air Properties Dataset

**Figure 4:**
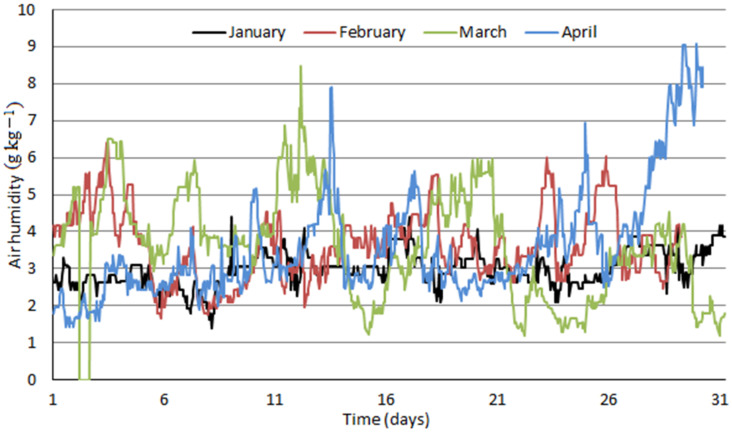
Hourly averages of air humidity in Košice—Center from January 2020 to April 2020.

**Figure 5:**
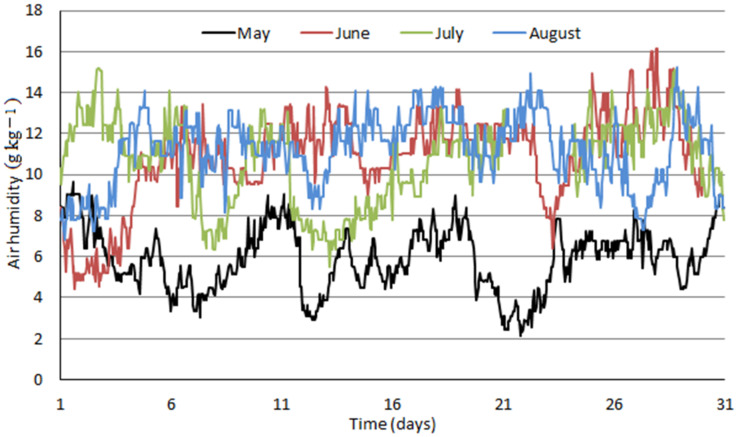
Hourly averages of air humidity in Košice—Center from May 2020 to August 2020.

**Figure 6:**
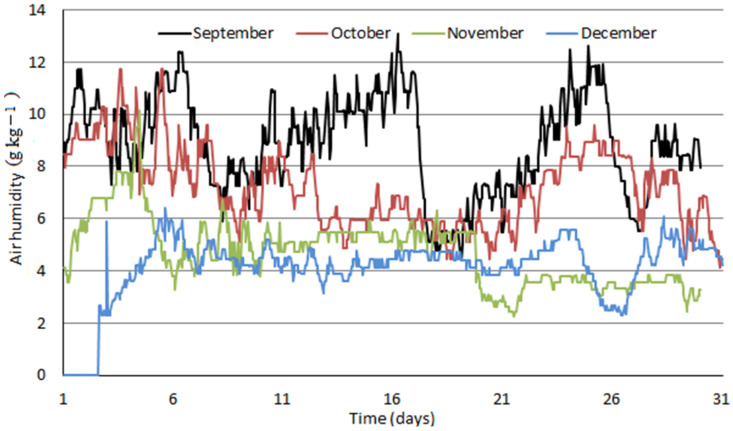
Hourly averages of air humidity in Košice—Center from September 2020 to December 2020.

Air properties dataset comprised a period from January 2020 to December 2020. It included hourly average values of atmospheric pressure, temperature, and relative humidity for location Košice—Center: altitude: 213 m above sea level, northern latitude: 48°43′41″, eastern longitude: 21°15′54″, Slovakia. Measured data were recalculated to yield air humidity, Y, in grams of water vapor present in air per one kilogram of dry air and are plotted in Figure 4, Figure 5 and Figure 6 as monthly data series.

Figure 4, Figure 5 and Figure 6 clearly distinguish between winter and summer months in terms of air humidity. Values around 2 to 4 g kg^−1^ are typical for winter months; air humidity increases quickly within one or two months to typical summer values of 10 to 14 g kg^−1^. Fluctuating humidity values affect the distribution of water removal between compressed air intercoolers and final adsorbers which is further influenced by enhanced air cooling by ACH proposed in this study.

### 2.6. Economic Calculations

For economic analysis, approximative investment costs of additional process equipment—ACH and five heat exchangers—were estimated. Approximate cost of ACH was obtained from available technical documentation [68] of real absorption coolers. The size of reference ACH is characterized by “cooling capacity”, which unit is equal to unit USRT (U.S. refrigeration ton). Additionally, model ACH obtained from technical documentation [63] are characterized by unit USRT, $USRT_{model}$, which is very close to the USRT of reference absorption coolers, $USRT_{ref}$ and, thus, enables linear cost recalculation without significant deviation from the generally used power law (cost dependence on specific equipment size to the power of 0.6 to 0.8). The cost of model ACH, $Cost_{model}$, can be estimated using Equation (2).

(2) $$Cost_{model}=\frac{USRT_{model}}{USRT_{ref}}\;Cost_{ref}$$

Calculated cost of model ACH is for 2016; its actual cost for 2020, $Cost_{2020}$, was calculated via the CEPCI index (Chemical Engineering Plant Cost Index), Equation (3), adopting the calculation procedure used in reference cost engineering literature [69]. An overview of parameters for model ACH investment cost estimation is presented in Table 1.

(3) $$Cost_{2020}=\frac{CEPCI_{2020}}{CEPCI_{2016}}\;Cost_{2016}$$

Investment cost of heat exchangers (additional coolers) E0, E6, E8, E10, and E12 was estimated by available cost curve dependent on the heat transfer area obtained from available literature [73]. Therefore, approximative heat transfer areas of additional model heat exchangers were estimated by solving the overall heat transfer Equation (4).

(4) $$\overset{\cdot }{Q}=U\;A\;\Delta T_{ls}$$

where U is the overall heat transfer coefficient, which is the function of fluid properties, material composition of the heat exchanger and the flow geometry with the estimated value of 100 W/m^2^/K [74]; $\Delta T_{ls}$ is the log-mean temperature difference calculated by Equation (5), where $t_{air,in},\;t_{air,out}$ are inlet and outlet temperatures of air and $t_{w,in},\;t_{w,out}$ are inlet and outlet temperatures of water.

(5) $$\Delta T_{ls}=\frac{\left(t_{air,in}-t_{w,out}\right)-\left(t_{air,out}-t_{w,in}\right)}{ln\frac{\left(t_{air,in}-t_{w,out}\right)}{\left(t_{air,out}-t_{w,in}\right)}}$$

Since the heat exchanger duty, $\overset{\cdot }{Q}$, is a known parameter, the only unknown parameter in Equation (5) is the heat transfer area. Final values of heat transfer area and cost of heat exchangers are provided in Table 2. 

Total estimated purchased cost of ACH and heat exchangers is EUR 5.41 mil. To calculate total investment cost, direct and indirect costs had to be added [76]. Total investment cost after adding direct and indirect cost to the purchase cost of process equipment is shown in Table 3, following the cost engineering guidelines in Peters et al. [69]. The resulting total investment cost to key equipment cost of 3.83 is somewhat lower than the typical values of 4 to 6 reported in [69] due to the proposed technology change and the already available infrastructure and is thus somewhat lower than an analogous green field investment. No operating costs other than electric energy consumption for ACH auxiliaries (pumps, fans, etc.), based on vendor documentation [63] were considered.

### 2.7. Model Industrial Thermal Power Plant

**Figure A2:**
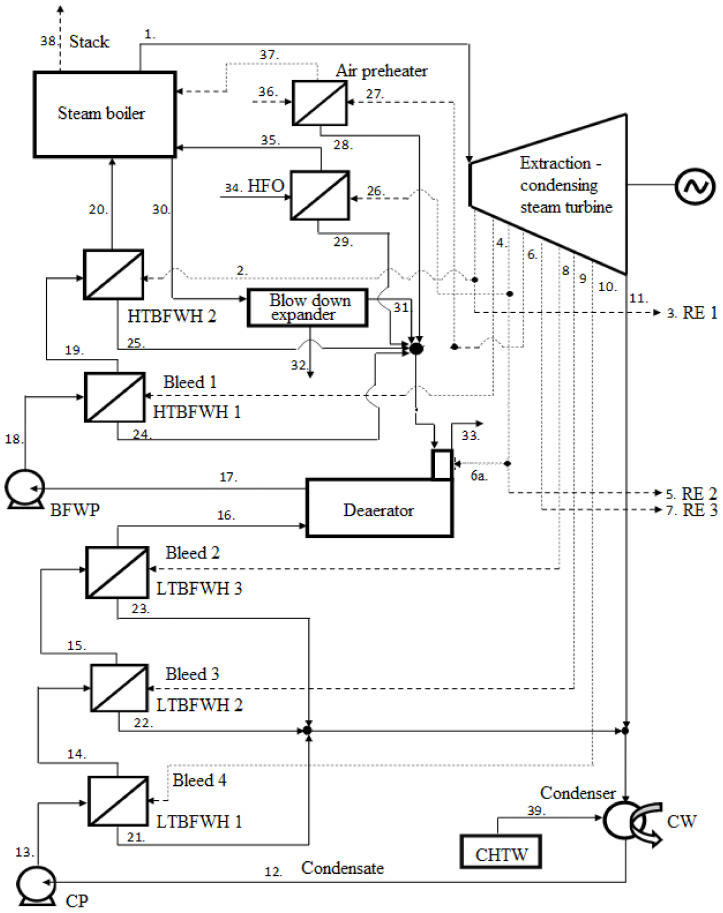
Model industrial thermal power plant. BFWP—boiler feedwater pump; CP—condensate pump; CHTW—chemically treated water; CW—cooling water; HFO—heavy fuel oil; HTBFWH—high-temperature boiler feedwater heater; LTBFWH—low-temperature boiler feedwater heater; RE—regulated extraction. Source: own elaboration.

An industrial thermal Clausius-Rankine power plant model was set up (Appendix C, Figure A2), mirroring a real plant operating in SLOVNAFT refinery [77]. 

Automated monitoring system (AMS) outputs in forms of yearly reports are available on SLOVNAFT websites: [78]: years 2012–2016 and [79]: year 2018, comprising the amounts of produced greenhouse gas emissions and particulate matter in the industrial thermal power plant as well as in refinery´s production units. An excerpt of these data is provided in Table 4.

The given data were used to calculate specific emissions of carbon monoxide, nitrogen oxides, and sulfur oxides per one ton of emitted carbon dioxide and the values are shown in Table 5. A reasonable value of carbon dioxide produced per unit of fuel combusted can obtained from fuel combustion stoichiometry. Thus, data in Table 5 served to calculate greenhouse gases’ (other than CO_2_) emission from a model industrial thermal power plant.

## 3. Results and Discussion

### 3.1. ASU Model Verification

Key results of mathematical model verification and comparison with model design simulation data are presented in Appendix D, Table A9, Table A10 and Table A11.

Verification of the developed mathematical model via simulation show very good correspondence with data of the selected basic model [36], with key variables values differences below 1%. The required quality and quantity of product streams are achieved. For further modifications and simulations, model parameters in the cryogenic section are kept the same.

### 3.2. Results of ASU Design and Operation Improvement by Absorption Cooling

#### 3.2.1. Exploitation of Internal Waste Heat for Cold Production

Key simulation results of the compression section process equipment obtained from the mathematical model including absorption coolers exploiting internal waste heat and their comparison with results obtained from a basic mathematical model are presented in Table 6, Table 7, Table 8, Table 9 and Table 10.

Compression ratio and mass flow of compressed air are affected by ACH implementation both in compressor K2 (Table 6) and compressor K3 (Table 7). As for compressor K2, the increase in the compression ratio overweighs the effect of a slight air mass flow decrease as well as that of decreased inlet air temperature, which results in an almost 1.5 MW power consumption increase (+7.5%). In compressor K3, the compression ratio, compressed air mass flow, as well as inlet air temperature decrease after ACH implementation, leading to an almost 10% decrease in its power consumption. However, since its nominal power input is lower than 10% of that of compressor K2, the overall effect on power consumption for air compression in all compressors together is negative.

The change in compressed air mass flow after ACH implementation is documented in Table 8, Table 9 and Table 10 showing the redistribution of water vapor removal from air. While water vapor predominantly passes through the compression section and is removed in absorbers in the base case, its large portion is removed already in the compression section after ACH implementation (around 4 of total 5 t h^−1^, see Table 8) due to absorption cooling to a significantly lower temperature, thus a large share of water vapor is condensed and removed in separators F1 to F3. As shown in Table 9 and Table 10, larger adsorber load caused by much higher water adsorption rate in both adsorbers in the base case led to a substantially higher power consumption for their regeneration, with a calculated difference of almost +2 MW in case of A1 and of over 0.5 MW in case of A2. Therefore, implementation of ACHs results in an increased compressor load while that needed for adsorbers regeneration is cut down. Air pressure losses have a more significant impact on compression power consumption than the decreased inlet air temperature achieved by ACH implementation. Thus, air pressure loss in heat exchangers is the most important parameter, crucial to energy feasibility of ACH implementation. Air humidity effect on the power consumption for adsorbers regeneration, etc., has a profound impact on achievable energy savings. Thus, both parameters are examined in detail later.

All modeled cooling towers operate with the same model parameters. Results of representative cooling tower C1 simulation are presented in Appendix E, Table A12. Implementation of cooling towers into the developed mathematical model ensures the required inlet process temperature of cooling water to be achieved. Further adjustments of cooling tower model parameters are not needed. 

#### 3.2.2. Exploitation of External Waste Heat for Cold Production

The key results of this analysis are the results of the compressor K1 simulation, which are presented in Table 11.

Similarly, as in the model of ASU with ACH utilizing its own waste heat from the compression section, compressed air pressure loss in the modeled heat exchangers causes a significant increase in compressor power consumption although the inlet air temperature decreases by 10 °C. Results show that the proposed compression section improvements are infeasible at the model parameters assumed. 

### 3.3. Impact of Air Pressure Loss in Heat Exhangers

Model parameter—air pressure loss (10 kPa)—specified in all modeled heat exchangers causes significant pressure loss in the compression section, which has to be compensated by increasing the compression ratio in compressors, thus leading to significant compression work increase. Based on available technical documentation for real air–water coolers [80], the approximate real value of compressed air pressure loss in water coolers is a fraction of the inlet air pressure rather than a constant value. 

Thus, three representative situations are evaluated. The pressure loss of compressed air in all modeled heat exchangers is specified to be 0, 1, and 2% of inlet air pressure. Modification of the air pressure loss model parameter is performed for both the modified and basic mathematical model and the effect of air pressure loss on compression energy consumption is assessed by comparing the results of these mathematical models. The main monitored parameters include compressor power input and compression ratio. Results of models considering 1 and 2% air pressure loss in heat exchangers are shown in Table 12. This case study confirms the significant effect of air pressure loss on compression work. The electrical power input savings for models with zero pressure loss in all modeled heat exchangers equals approximately 2.18 MW. Table 12 shows that air pressure loss in heat exchangers of 2% of inlet air pressure results in only negligible electrical power savings for compression. With each air pressure loss decrease by 1%, the value of annual electrical power savings in the compression section increases by 9500 MWh.

### 3.4. Impact of Variable Ambient Air Humidity

The results of annual electrical power savings calculation for adsorber regeneration based on Equation (1) and constant ambient air properties assumed in [36] are provided in Appendix E, Table A13. The calculated total annual electrical power savings value is valid only for one model situation—constant value of air humidity during the year. Since air humidity is a variable parameter and its value depends on weather, season, location and other parameters, annual electrical power savings shown in Table A13 has to be recalculated employing ambient air properties dataset for Košice-Center location. 

In the model including absorption coolers, calculated value of air humidity in the inlet of adsorbers equals to 1.54 g kg^−1^ of dry air. In case of higher air humidity values, excess water in compressed air condenses in the compression section before entering the adsorbers and the inlet air humidity value does not change. As it results from Figure 4, Figure 5 and Figure 6, an air humidity value of 1.54 g kg^−1^ and lower occurs only very infrequently. Therefore, a constant value of electrical power input for adsorber regeneration can be assumed in this model.

**Figure 7:**
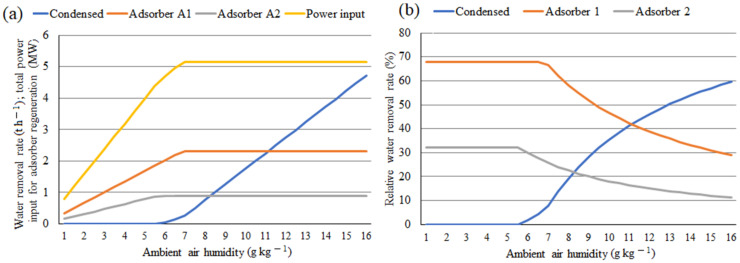
(**a**,**b**) Water removal rate and total power input for adsorber regeneration (**a**) and water removal rate distribution (**b**) as a function of ambient air humidity for model without absorption coolers. Condensed = water condensed in compression section intercoolers and coolers; Adsorber A1 = water removed in adsorber A1; Adsorber A2 = water removed in adsorber A2.

In the model without absorption coolers, calculated values of air humidity at the inlet of adsorbers A1 (6.48 g kg^−1^) and A2 (5.63 g kg^−1^) are significantly higher than in the model with absorption coolers. Figure 7 shows that in case of higher air humidity values, excess water condenses in the compression section. However, air humidity values below the mentioned values (6.48 and 5.63 g kg^−1^) results in smaller amount of water adsorbed and, subsequently, in lower power consumption for adsorbers regeneration. This is apparent from Figure 7a,b showing the calculated water removal by compressed air intercooling and by adsorption and distribution. Its impact on the required power input for adsorber regeneration can also be observed increasing linearly with air humidity up to 5.63 g kg^−1^, which represents saturated air at the inlet of adsorber A2. With still increasing air humidity, power consumption for adsorber A2 regeneration does not change any more, as the air at its inlet is already saturated and excess water vapor is removed in the compression section, while that for A1 further increases. As the power consumption for A1 regeneration is much higher than that for A2, total power consumption increase for regeneration decreases only slightly. After reaching air humidity of 6.48 g kg^−1^, saturated air conditions are achieved at adsorber A1 inlet, i.e., maximal adsorber load accompanied by maximal power consumption for the regeneration of both adsorbers are reached and do not change with further air humidity increase as excess water vapor is condensed in the compression section.

By processing the ambient air dataset, percentages of time during 2020 with air humidity values lower and higher than 6.48 and 5.63 g kg^−1^, respectively, are estimated. Moreover, the average air humidity is calculated for the fraction of the year with lower air humidity. This value is subsequently used to calculate a new average electrical power input for adsorber regeneration in the model without absorption coolers, as presented in Appendix E, Table A14.

The final value of recalculated annual electrical power savings for adsorber regeneration is presented in Table 13 and it is used for further calculations related to economical assessment. Compared to the value of electrical power savings presented in Table A13, the recalculated value of electrical power savings for model location Košice-Center is by 10,570 MWh lower. Nevertheless, compared to the annual power consumption of all three compressors together plus power input for adsorbers regeneration, amounting to 353 GWh per year, the achievable power consumption reduction is 6.7%.

### 3.5. Economic Parameters

Feasibility of the proposed investment—implementation of absorption coolers and additional heat exchangers to the basic cryogenic air separation model—is evaluated through a simple payback period (SPBP) using Equation (6).

(6) $$SPBP=\frac{Total\;investment\;cost}{Annual\;cash\;flow}$$

Total investment cost (EUR 20.7 mil) is estimated in part 2.6 Economic Calculations. Annual cash flow results from electrical power savings for compressors operation and for adsorbers regeneration. 

Power input savings for compressors K1, K2, and K3 depend on the percentage of air pressure loss in the modeled heat exchangers; final savings are presented in Table 14. Annual power consumption for ACH pumps is subtracted from the total annual electrical power savings obtained from available technical documentation for a real ACH [63].

**Figure 8:**
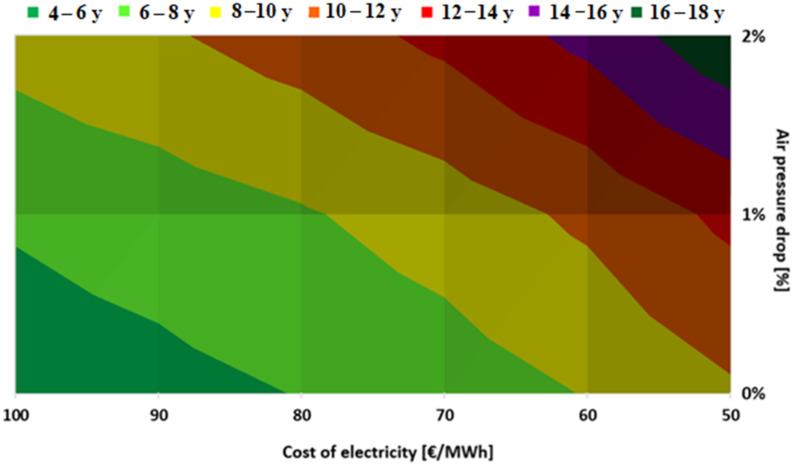
Simple payback period values in years as a function of electricity price and air pressure loss.

Results in Table 14 indicate significant influence of varying air pressure loss in the modeled heat exchangers on electrical power savings. Under theoretical conditions—zero air pressure loss—the highest electrical power savings can be achieved. With each air pressure loss increase by 1%, the electrical power savings value is reduced by 9500 MWh per year. Annual cash flow is obtained by multiplying the total annual power savings in Table 14 by the cost of electricity assumed in the range of EUR 50–100/MWh^−1^. Results of the sensitivity analysis are provided in Figure 8. As can be seen, simple payback period values vary from 4 to 18 years, with both air pressure loss and electricity price being important.

It should, however, be remembered that the price of carbon dioxide emissions increased rapidly over the last two years, currently exceeding EUR 55 t^−1^ [81] and it is expected to reach EUR 150 t^−1^ until 2050 [52], which is partly reflected in the currently growing electricity prices. Continuation of this trend significantly increases the economic feasibility of the proposed ASU performance improvement and reduces the simple payback period to 5 years even in case of a 2% air pressure loss in the heat exchangers. Simple recalculation of the above figures yields achievable reduction of specific oxygen production cost of EUR 2–4.2 t^−1^ which represents 3 to 5% of typical oxygen production cost [30].

### 3.6. Achievable Reduction of Greenhouse Gas Emissions

The following thermal power plant parameters are obtained, expressed per 1 MWh of net produced electricity: steam production in steam boiler: 4.537 t; fuel consumption: 0.2904 t; CO_2_ emissions: 0.926 t. The resulting net power production efficiency of 30.6% is somewhat lower than efficiencies of up to 40 (45)% reached in large power plants [82] because of the conservative design and parameters of the considered plant with lower live steam parameters, lower number of boiler feedwater heaters, and absence of steam reheating. Variny et al. [83] reported a value of specific net power production of 3 MWh per 1 ton of combusted fuel in an industrial steam power plant, which agrees well with the obtained specific fuel consumption of 0.2904 t per 1 MWh of net power production (3.44 MWh per ton of fuel).

The obtained specific CO_2_ emissions of slightly over 0.9 t MWh^−1^ are comparable with those from >100 MW range subcritical coal-fired power plants [84], or oxyfuel power plants [39] with the lower net power production efficiency impact somewhat counterbalanced by lower carbon content in fuel compared to common coal. SO_x_ and NO_x_ emissions listed in Table 5 can be recalculated to values per MWh of net produced electricity, yielding 0.707 kg MWh^−1^ for SO_x_ and 1.132 kg MWh^−1^ for NO_x_, respectively. They are significantly higher than values reported by Campbell et al. [84] for a large coal-fired power plant, reaching 0.2 kg MWh^−1^ for SO_x_ and 0.472 kg MWh^−1^ for NO_x_, respectively. However, Strachan and Farrell [85] listed specific emissions of greenhouse gases from various power production technologies, including a coal-fired power plant with three- to five-fold higher values for both SO_x_ and NO_x_ than those obtained in this study, while the corresponding CO_2_ emission factor of 0.965 t MWh^−1^ presented almost equals with that calculated in this study. Table 15 shows a comparison of the CO_2_ emission factors reported in further relevant literature and the resulting achievable cut in annual CO_2_ emissions. In a similar manner, Table 16 lists the expected NO_x_ and SO_x_ emissions decrease. For comparison, both Table 15 and Table 16 contain emission factors representing an energy mix of Slovenské elektrárne, a.s., the major electric energy producer in Slovak Republic, adopted from [83]. Slovenské elektrárne, a.s. produce a large share of electricity in nuclear plants and hydro power plants and are thus considered an environmentally friendly electricity producer.

Emission factors for CO_2_ reported in various studies and calculated in this study range between 0.7 to over 0.9 t MWh^−1^, so the expected cut in its emissions is between 23 to 31 kilotons per year and between 16 to 22 kilotons per year for 1 and 2% air pressure loss in the heat exchangers, respectively. Thus, the uncertainty regarding the pressure loss value in the heat exchangers has a comparable effect on CO_2_ emissions’ reduction as the fossil fuel-based emission factor variability. If a more environmentally friendly energy mix is considered, CO_2_ emission reduction due to ACH implementation can be reduced by around 80%. This is in line with the general understanding that increased share of renewables in the consumed electric energy reduces the environmental benefit of its consumption reduction.

However, as it results from Table 16, there is a much wider variability in NO_x_ and SO_x_ emission factors yielding a very wide range of possible emission reduction of these pollutants as CO_2_ emissions depend on fuel composition solely (if complete combustion can be assumed) while those of CO, NO_x_, and SO_x_ depend also on process conditions and parameters [87] and on the way how (and if) flue gas is cleaned before being emitted to the atmosphere [88]. Implementation of emission factors from energy mix [83] increases the range of possible emission reduction further, especially in case on NO_x_ emissions where it amounts to almost 80%. In case of SO_x_, a further reduction of the annually released amount of around 50% (9.2 vs. 16.7 tons per annum) can be seen in Table 16. The effect of air pressure loss in the heat exchangers on the resulting emissions’ reduction is almost negligible in comparison. 

From a quantitative point of view, it can be concluded that the achievable annual power consumption cut in the considered air separation units between 23 to 33 GWh (6.7 to 9.4%) translated into annual CO_2_ emissions’ reduction by 16 to 31 kilotons and reduction of annual emissions of other major gaseous pollutants by up to 187 tons.

## 4. Conclusions

The present study provides a complex economic–energetic–environmental assessment of a model cryogenic ASU design’s improvement by air pre-cooling and intercooling, using absorption coolers powered by waste heat from the compression section. Air humidity and air pressure loss in heat exchangers were identified as two key factors affecting energy consumption reduction when absorption coolers are implemented. Hourly variations of ambient air humidity were considered and water vapor removal distribution in the compression section and air dryers for the location Košice (Central Europe) was assessed, resulting in a realistic estimate of the corresponding power consumption for air dryer regeneration. 

Achievable power savings of 6.7 to 9.4% are consistent with those presented in similar recent studies. For a model ASU processing 500 t h^−1^ of ambient air, power savings amounted to 23 to 33 GWh; the given interval results from the uncertainty due to air pressure loss in the newly added heat exchangers. For very humid climate locations, this value increased by yet another 10 GWh per annum due to higher power savings achieved in air dryer regeneration. Within a reasonable range of air pressure loss in heat exchangers of 1 to 2%, assumed technology, location in Košice, and electricity price interval of EUR 50–100/MWh^−1^, simple payback period of 4 to 18 years was estimated with total investment costs amounting to over EUR 20 mil.

Calculation of the related greenhouse gases emissions’ reduction included both our own heavy oil-fueled thermal power plant model and emission factors reported in the literature for given or similar fuels. As a result, a wide range of emission factors of major air pollutants was obtained, which is a far more realistic approach than a single value for every pollutant. The related final values of annual emission reduction potential in fossil fuel-based power production amounted to CO_2_ 16 to 30 kilotons, CO 0.3 to 2.3 tons, SO_x_ 4.7 to 187 tons, and NO_x_ 11 to 56 tons. If a renewable resources rich energy mix was considered for power production, the corresponding pollutant emissions’ reduction values were reduced by 50 to 80%, depending on the considered pollutant.

It can be concluded that the chosen approach to design and operation improvement of cryogenic air separation units proved to be feasible, delivering power consumption and greenhouse gases emissions’ reduction with an acceptable simple payback period. Implementing the proposed changes in existing cryogenic air separation units has the potential to make oxygen production more efficient and sustainable.

## Figures and Tables

**Table 1:** Estimation of ACH investment costs. ACH: absorption cooler, CEPCI: Chemical Engineering Plant Cost Index, USRT: U. S. Refrigeration Ton.

Parameter	ACH 0	ACH 1	ACH 2
USRT of referential ACH [68]	400	1320	1320
Specific cost of referential ACH (2016) (USD USRT^−1^) [68]	2300	1800	1800
USRT of model ACH [63]	375	1125	1350
Specific cost of model ACH (2016) (USD USRT^−1^)	1841	1534	2156
Cost of model ACH (2016) (USD)	808,594	1,725,852	2,485,227
Exchange rate (USD EUR^−1^) [70]	0.83		
CEPCI index (2016) [71]	541.7		
CEPCI index (2020) [72]	668.0		
Cost of model ACH (2020) (EUR)	827,610	1,766,442	2,543,676
Total cost of ACH (2020) (EUR)	5,150,000		

**Table 2:** Estimation of heat exchangers investment costs.

Heat Exchanger/Parameter	E0	E6	E8	E10	E12
$\overset{\cdot }{Q}\;$ (MW)	1336.2	6392.5	5190.8	5327.4	4324.9
U (W m^−2^ K^−1^)	100				
ΔT (°C)	9.8	30.9	16.0	45.8	15.3
A (m^2^)	1362.4	2068.4	3242.9	1164.3	2826.1
*A* with margin (m^2^)	1500	2500	3500	1500	3000
Cost (1998) (USD) [73]	25,000	40,000	50,000	25,000	45,000
Exchange rate (USD EUR^−1^) [70]	0.83				
CEPCI index (1998) [75]	389.5				
CEPCI index (2020) [72]	668.0				
Cost (2020) (EUR)	35,586	56,938	71,113	35,586	64,056
Total investment cost (EUR)	264,000				

**Table 3:** Total investment cost estimation [69].

Purchase Cost of Equipment (EUR mil.)		5.41 (100%)	
Direct Cost		Indirect Cost	
Installation of equipment	40%	Projecting and control	33%
Measurement and regulation	35%	Building costs	20%
Pipes	70%	Legalization costs	4%
Land consolidation	10%	Payments to contractors	20%
Services	10%	Reserves	30%
		Circulating capital	10%
Total direct and indirect costs		282%	
Total investment costs (EUR mil.)		20.7 (383%)	

**Table 4:** Selected data on greenhouse gas emissions from thermal power plant based on [78,79].

Emissions (t)	Year (Days of Validated AMS Operation and Data Recording)				
	2013 (249)	2014 (242)	2015 (72)	2016 (316)	2018 (Not Provided)
CO_2_	318,256	347,916	128,889	517,115	776,743
CO	4.108	12.696	1.311	2.828	10.278
NO_x_	336.851	393.087	126.009	531.929	1165.025
SO_x_	142.791	226.096	74.696	444.756	705.554

**Table 5:** Average specific emissions of pollutants in grams per ton of produced CO_2_.

Pollutant	Average Specific Emissions in Grams per Ton of Produced CO_2_
CO	15.1
NO_x_	1222
SO_x_	763

**Table 6:** Comparison of key parameters related to compressor K2 obtained by basic ASU model and by model comprising ACH.

Compressor K2						
	Model with ACH			Basic Mathematical Model		
Stream	m (t h^−1^)	t (°C)	P (kPa)	m (t h^−1^)	t (°C)	P (kPa)
S4	497.3	16.7	220	500	40.0	240
S6	497.3	156.9	630	500	168.9	600
Power input (kW)	19,620			18,252		

**Table 7:** Comparison of key parameters related to compressor K3 obtained by basic ASU model and by model comprising ACH.

Compressor K3						
	Model with ACH			Basic Mathematical Model		
Stream	m (t h^−1^)	t (°C)	P (kPa)	m (t h^−1^)	t (°C)	P (kPa)
S14	158.7	16.2	600	159.5	40.0	590
S16	158.7	44.2	760	159.5	70.7	750
Power input (kW)	1241			1377		

**Table 8:** Comparison of key parameters related to flash separators obtained by basic ASU model and by model comprising ACH.

Flash Separators F1, F2, F3, F4						
	Model with ACH			Basic Mathematical Model		
Stream	m (kg h^−1^)	t (°C)	P (kPa)	m (kg h^−1^)	t (°C)	P (kPa)
S5 (F1)	2694.1	16.7	220	0	40	240
S15 (F2)	443.8	16.2	600	244.4	40	590
S11 (F3)	943.6	16.2	600	519.2	40	590
S19 (F4)	0	-	-	252.2	40	740

**Table 9:** Comparison of key parameters related to adsorber A1 obtained by basic ASU model and by model comprising ACH. Q_reg_: power input for adsorber regeneration.

Adsorber A1						
	Model with ACH			Basic Mathematical Model		
Stream	m (t h^−1^)	t (°C)	P (kPa)	m (t h^−1^)	t (°C)	P (kPa)
S10	337.2	16.2	600	339	40	590
S12A	336.5	20.9	590	336.5	60.5	580
H_2_O (kg h^−1^)	542.9			2498.4		
Q_reg_ (kW)	452.4			1952		

**Table 10:** Comparison of key parameters related to adsorber A2 obtained by basic ASU model and by model comprising ACH.

Adsorber A2						
	Model with ACH			Basic Mathematical Model		
Stream	m (t h^−1^)	t (°C)	P (kPa)	m (t h^−1^)	t (°C)	P (kPa)
S10	158.6	35.0	750	159.3	40.0	740
S12A	158.4	39.7	740	158.4	56.5	730
H_2_O (kg h^−1^)	255.1			886.6		
Q_reg_ (kW)	212.6			738.8		

**Table 11:** Comparison of key parameters related to compressor K1 obtained by basic ASU model and by model comprising ACH.

Compressor K1						
	Model with ACH			Basic Mathematical Model		
Stream	m (t h^−1^)	t (°C)	P (kPa)	m (t h^−1^)	t (°C)	P (kPa)
S1	500	15.5	90	500	25.0	100
S2	500	159.2	250	500	147.9	250
Power input (kW)	20,297			17,381		

**Table 12:** Key model parameters sensitivity to air pressure loss in heat exchangers. Δ: Difference.

Air Pressure Loss	2%		1%	
Compressor	Model without ACH (MW)	Model with ACH (MW)	Model without ACH (MW)	Model with ACH (MW)
K1	17.38	17.26	17.38	17.05
K2	18.23	18.46	17.78	17.13
K3	1.394	1.273	1.339	1.222
Δ power K1 (MW)	0.120		0.33	
Δ power K2 (MW)	−0.230		0.65	
Δ power K3 (MW)	0.121		0.117	
Total power savings (MW; %)	0.011; negligible		1.097; 3.0	

**Table 13:** Recalculated power input savings in model with ACH compared to basic model (without ACH).

Parameter	A1	A2
Recalculated electrical power input for adsorber regeneration (model without ACH) (MW)	2.767	1.167
Electrical power input for adsorber regeneration (model with ACH) (MW)	0.836	0.393
Recalculated electrical power input savings (MW)	1.931	0.773
Recalculated annual (8760 h per year) electrical power savings for adsorber regeneration (MWh)	16,916	6775
Total recalculated annual electrical power savings for adsorbers regeneration (MWh)	23,691	

**Table 14:** Total annual power savings calculation resulting from ACH implementation.

% of Air Pressure Loss	2%	1%	0%
Electrical power input savings for compression (MW)	0.011	1.097	2.183
Annual electrical power savings for compression (MWh)	96.4	9609.7	19,123.1
Annual electrical power savings for adsorber regeneration (MWh)	23,691		
Annual electrical power consumption for ACH pumps (MWh)	−200		
Total annual power savings (MWh)	23,588	33,101	42,614

**Table 15:** Decrease in annual emissions of CO_2_ resulting from ACH implementation.

Study/ Value	This Study	[86]	[54]	[57]	[31]	[83]
Fuel	HFO	Heating oil	Heavy oil	Heavy oil	Coal	Energy mix
Emission factor (t MWh^−1^)	0.926	0.778 (min. 0.731, max. 0.857)	0.72	0.802 old plant; 0.702 new plant	0.86 conventional plant; 0.743 ultra-supercritical plant	0.136
Annual CO_2_ emissions decrease (Table 14, air pressure loss 1%) (t)	30,652	24,197 to 28,368	23,833	23,237 to 26,574	24,594 to 28,467	4502
Annual CO_2_ emissions decrease (Table 14, air pressure loss 2%) (t)	21,842	17,243 to 20,215	16,983	16,559 to 18,918	17,526 to 20,286	3208

**Table 16:** Decrease in annual emissions of other major gaseous pollutants resulting from ACH implementation.

Study/ Value	This Study			Campbell et al. [84] (Coal Power Plant)		Strachan and Farrell [85] (Coal Power Plant)			Variny et al. [83] (Electric Energy Mix of Slovenské Elektrárne, a.s.)		
Pollutant	CO	SO_x_	NO_x_	CO	SO_x_	NO_x_	SO_x_	NO_x_	CO	SO_x_	NO_x_
Emission factor (kg MWh^−1^)	0.014	0.707	1.132	0.07	5.64	1.7	0.2	0.459	0.061	0.392	0.107
Annual emissions decrease (Table 14, air pressure loss 1%) (t)	0.5	23.4	37.5	2.3	186.7	56.3	6.6	15.2	2.0	13.0	3.5
Annual emissions decrease (Table 14, air pressure loss 2%) (t)	0.3	16.7	26.7	1.7	133.0	40.1	4.7	10.8	1.4	9.2	2.5

## Data Availability

All data obtained by calculations and analyses are listed directly in this study.

## Appendix A

**Table A1:** Inlet air parameters and composition [36].

Parameter	Value
Mass flow (t h^−1^)	500
Temperature (°C)	25
Pressure (kPa)	100
Composition (mol.)	Value
Nitrogen	0.7686
Oxygen	0.2062
Argon	0.0092
Carbon dioxide	0.0003
Water vapor	0.0156

**Table A2:** Inlet cooling water parameters [29].

Parameter	Value
Mass flow (t h^−1^)	4700
Temperature (°C)	25
Pressure (kPa)	100

**Table A3:** Model equipment input parameters. HPC: high-pressure column; LPC: low-pressure column.

Process Equipment	Model	Input Parameters
Compressor	Compr	Inlet pressure of air Isentropic efficiency
Heat exchanger	HeatX	Outlet temperature of air Pressure loss
Multi-stream heat exchanger	MHeatX	Outlet temperature of streams Pressure loss
Flash separator	Flash2	Pressure
Adsorber	Sep	Pressure
Distillation column	RadFrac (without condenser/reboiler)	Pressure in condenser and reboiler, Pressure loss Mass flow of distillate (HPC) Reboiler duty (LPC) Number of stages Feed stages
Pump	Pump	Outlet pressure of stream Isentropic efficiency

## Appendix B

**Table A4:** Parameters of absorption cooler (ACH) placed after first compression stage. COP: coefficient of performance; USRT: U.S. refrigeration ton. Source: [63].

Parameter	Value
Model of ACH	Hot-water/135
USRT (U.S. refrigeration ton)	1350
COP	0.8
Hot water	
Inlet temperature to generator (°C)	95
Outlet temperature from generator (°C)	72
Mass flow (t h^−1^)	219.2
Chilled water	
Inlet temperature to evaporator (°C)	13
Outlet temperature from evaporator (°C)	8
Volumetric flow (m^3^ h^−1^)	816.5
Cooling water	
Inlet temperature (°C)	31
Outlet temperature (°C)	36.5
Volumetric flow (m^3^ h^−1^)	1658.6

**Table A5:** Parameters of ACH placed after second compression stage. Source: [63].

Parameter	Value
Model of ACH	Hot-water/113
USRT (U.S. refrigeration ton)	1125
COP	0.8
Hot water	
Inlet temperature to generator (°C)	95
Outlet temperature from generator (°C)	72
Mass flow (t h^−1^)	182.6
Chilled water	
Inlet temperature to evaporator (°C)	13
Outlet temperature from evaporator (°C)	8
Volumetric flow (m^3^ h^−1^)	680.4
Cooling water	
Inlet temperature (°C)	31
Outlet temperature (°C)	36.5
Volumetric flow (m^3^ h^−1^)	1382.2

**Table A6:** Parameters of cooling towers. Source: [64].

Parameter	Value
Type of cooling tower	Fan
Model of cooling tower	12/70 ZB
Number of fans	12
Maximum cooling range (°C)	7
Wet bulb temperature (°C)	21
Volumetric flow of air (m^3^ h^−1^)	504,000
Nominal mass flow of cooling water (t h^−1^)	1225
Minimum/Maximum mass flow of cooling water (t h^−1^)	276/1476

**Table A7:** Parameters of cooling towers used for modeling in Aspen Plus.

Parameter	Value
Pressure (kPa)	100
Number of equilibrium stages	10
Mass flow of cooling water (t h^−1^)	1175
Inlet temperature of cooling tower (°C)	32.6
Volumetric flow of air (m^3^ h^−1^)	504,000
Inlet temperature of air (°C)	25

**Table A8:** Parameters of ACH exploiting external heat. Source: [63].

Parameter	Value
Model of ACH	Hot-water/038
USRT (U.S. refrigeration ton)	375
COP	0.8
Chilled water	
Inlet temperature to evaporator (°C)	13
Outlet temperature from evaporator (°C)	8
Volumetric flow (m^3^ h^−1^)	226.8
Cooling water	
Inlet temperature (°C)	31
Outlet temperature (°C)	36.5
Volumetric flow (m^3^ h^−1^)	460.7

## Appendix C

The modeled industrial combined heat and power unit includes a very high-pressure steam boiler fueled by heavy fuel oil (HFO) and an extraction-condensing steam turbine, plus auxiliary equipment (Figure A2). It includes common power plant features such as air and fuel preheating and stepwise boiler feedwater heating [89,90] and it supplies the refinery with steam on three pressure levels. It has a spare condensing power production capacity of several 10^1^ MW which can be used to produce power for the ASU operation. Since 2012, a flue gas desulfurization unit (FGD) has been operated, reducing SO_x_ emissions by around 80% compared to the past [91]. The following assumptions were used in model construction:

- HFO composition in % wt.: carbon 87; hydrogen 12; sulfur 1; HFO lower heating value: 40.5 GJ t^−1^;
- Combustion air excess: 30%; preheated air temperature: 100 °C;
- Flue gas to stack (to FGD) temperature: 190 °C (margin of around 50 °C to minimize risk of low-temperature corrosion);
- Steam boiler blow down rate: 1.5%;
- Steam turbine inlet parameters: 9 MPa, 530 °C;
- Steam turbine isentropic efficiency: 85% in superheated steam region and 75% in wet steam region was used to calculate enthalpies of individual bleeds and regulated extractions in the water steam chart as recently demonstrated by Baressi et al. [92]; mechanical efficiency: 95%;
- Bleed pressure estimation: to keep a margin of 5 °C between bleed condensing temperature and water outlet temperature from water heater;
- Regulated extractions pressure: RE 1: 3.06 MPa; RE 2: 1 MPa; RE 3: 0.4 MPa;
- Temperature in turbine condenser: 30 °C;
- Stepwise condensate heating to 60, 90, 120, 150, 180, and 210 °C in low-temperature water heaters, in deaerator, and in high-temperature water heaters;
- Steam losses from deaerator: 6% of inlet steam;
- Internal power consumption: 5% of gross power production;
- Zero steam export from the power plant = condensing power production;

## Appendix D

**Table A9:** Model verification: compression and purification section.

Stream	This Study			Study [36]		
	m (t h^−1^)	t (°C)	P (kPa)	m (t h^−1^)	t (°C)	P (kPa)
S1	500	25	100	500	25	100
S2	500	147.9	250	500	146	250
S3–S4	500	40	240	500	40	240
S5	0	40	240	0	40	240
S6	500	168.9	600	500	168.9	600
S7	500	40.0	590	500	40.0	590
S8	160	40	590	160	40	590
S9	340	40	590	340	40	590
S10	339.0	40	590	339.0	40	590
S11	976	40	590	1000	40	590
S12	336.5	40	590	336.5	40	590
S13	2.5	40	590	2.5	40	590
S14	159.5	40.0	590	159.5	40.0	590
S15	459.3	40	590	500	40	590
S16	159.5	70.7	750	159.5	70.8	750
S17	159.5	40	740	159.5	40	740
S18	159.3	40	740	159.3	40	740
S19	215.7	40	740	200	40	740
S20	158.4	40	740	158.4	40	740
S20A	0.9	40	740	1.0	40	740
W2	4700	25.0	330	4700	25.0	330
W3	4700	27.6	320	4700	27.6	320
W4	4700	30.9	310	4700	30.9	310
W5	4700	31.1	300	4700	31.1	300

**Table A10:** Model verification: cryogenic section.

Stream	This Study			Study [36]		
	m (t h^−1^)	t (°C)	P (kPa)	m (t h^−1^)	t (°C)	P (kPa)
S21	158.4	−171.7	730	158.4	−171.7	730
S22	336.5	−160	580	336.5	−160	580
S23	158.4	−174.9	580	158.4	−174.9	580
S24	336.5	−160	580	336.5	−160	580
S25	199.1	−177.5	560	199.1	−177.5	560
S26	295.8	−173.3	580	295.8	−173.3	580
S27	199.1	−185.7	550	199.1	−185.7	550
S28	295.8	−180.6	570	295.8	−180.6	570
S29	199.1	−192.1	150	199.1	−192.1	150
S30	295.8	−189.0	150	295.8	−189.0	150
S31	380.4	−193.9	120	378.6	−194.1	120
S32	114.5	−179.3	150	116.3	−179.3	150
S33	114.4	−179.2	290	116.3	−179.2	480
S34	114.4	38.3	280	116.3	39.0	470
S35	380.4	−174.9	110	378.6	−175	110
S36	380.4	38.0	100	378.6	39.0	100

**Table A11:** Model verification: molar stream composition—HPC and LPC column.

Stream	This Study			Study [36]		
	N_2_	O_2_	Ar	N_2_	O_2_	Ar
S23	0.78	0.21	0.01	0.78	0.21	0.01
S24	0.78	0.21	0.01	0.78	0.21	0.01
S25	1.00	0.00	0.00	0.99	0.00	0.00
S26	0.63	0.36	0.01	0.63	0.36	0.01
S29	1.00	0.00	0.00	0.99	0.00	0.00
S30	0.63	0.36	0.01	0.63	0.36	0.01
S31	1.00	0.00	0.01	0.99	0.00	0.00
S32	-	0.99	0.01	0.00	0.99	0.00

## Appendix E

**Table A12:** Key operating parameters of cooling towers implemented in the model with ACH.

Stream	m (t h^−1^)	t (°C)	P (kPa)
W12	1175.1	33.0	100
S37 (m^3^ h^−1^)	504,000	25.0	100
W16	1161.6	25.3	100
S41	598.9	33.1	100

**Table A13:** Comparison of power consumption for adsorber regeneration in basic model (without ACH) and in the model with ACH.

Parameter (without ACH)	A1	A2
Inlet air humidity (g kg^−1^)	6.845	5.632
Mass flow of adsorbed water (kg h^−1^)	2304.7	892.4
Electrical power input for adsorber regeneration (MW)	3.706	1.435
**Parameter (with ACH)**	**A1**	**A2**
Inlet air humidity (g kg^−1^)	1.545	1.545
Mass flow of adsorbed water (kg h^−1^)	520.3	244.8
Electrical power input for adsorber regeneration (MW)	0.836	0.394
Electrical power input savings (MW)	2.869	1.041
Annual electrical power savings (MWh)	25,138.1	9123.2
Total annual electrical power savings (MWh)	34,261.3	

**Table A14:** Recalculation of power input for adsorber regeneration without ACH employing ambient air humidity dataset presented in Figure 4, Figure 5 and Figure 6.

Parameter	A1	A2
Calculated inlet air humidity (g kg^−1^)	6.845	5.632
Mass flow of adsorbed water (kg h^−1^)	2304.7	892.4
Calculated electrical power input for adsorber regeneration (MW)	3.706	1.435
Time of the year with higher measured air humidity (%)	38.5	46.5
Time of the year with lower measured air humidity (%)	61.5	53.5
Average air humidity for electrical power savings recalculation (g kg^−1^)	4.02	3.66
Calculated average mass flow of adsorbed water for time of the year with lower air humidity (kg h^−1^)	1356.3	580.7
Calculated average electrical power input for adsorber regeneration for time of the year with lower air humidity (MW)	2.181	0.934
Recalculated average electrical power input for adsorber regeneration (MW)	2.767	1.167
